# Supplementary material for: Leaf ethanolic extract of Etlingera hemesphaerica Blume mitigates defects in fetal anatomy and endochondral ossification due to mercuric chloride during the post-implantation period in Mus musculus
Source: PLoS One. 2021 Mar 10;16(3):e0247467. doi: 10.1371/journal.pone.0247467 (PMC7946197; doi:10.1371/journal.pone.0247467)
Supplement: Attachment — Submitted filename: Ruyani, Response to reviewer, Jan 4, 2021.docx [file pone.0247467.s001.docx]

| **No.** | **Editor and Reviewer's note** | **Our response** |
| --- | --- | --- |
| 1 | However, your Data Availability Statement in the online submission form still reads as follows:  "No" |  |
| 2 | If the minimal data set is located in your manuscript and/or Supporting Information files, please confirm whether we may update your Data Availability statement as follows:  “All relevant data are within the manuscript and its Supporting Information files.” |  |
| 4 | Further to previous requests we do need to ask you to address our requirement with regards to placing your tables in the manuscript rather than having them uploaded separately. | Our tables in the manuscript rather than having them uploaded separately. |
