# Supplementary material for: Leaf ethanolic extract of Etlingera hemesphaerica Blume mitigates defects in fetal anatomy and endochondral ossification due to mercuric chloride during the post-implantation period in Mus musculus
Source: PLoS One. 2021 Mar 10;16(3):e0247467. doi: 10.1371/journal.pone.0247467 (PMC7946197; doi:10.1371/journal.pone.0247467)
Supplement: Attachment — Submitted filename: Ruyani, Response to reviewer, Feb 4, 2021.docx [file pone.0247467.s002.docx]

| **No.** | **Editor and Reviewer's note** | **Our response** |
| --- | --- | --- |
| 1 | Both reviewers agree that the revised manuscript is a great improvement. Reviewer 1 wants to see that details about statistical methods are included in the manuscript. With this addition, the manuscript should be acceptable. I look forward to seeing this minor revision. | The data obtained from this study were generalized by the χ2 test of goodness of fit (Table 1) and by multiple comparation, and then the least significant difference (Table 3-6) [29]. |
